# Cannabis and Alcohol Use and Their Effects on Hematological and Biochemical Parameters: Evidence From the Accra Psychiatric Hospital, Ghana

**DOI:** 10.1002/hsr2.71908

**Published:** 2026-02-25

**Authors:** Felix Abekah Botchway, Prince Agyemang, Fati Sidi Yahaya, Emmanuel Baah‐Sackey, Christiana Anim, Emmanuel Abindau, Cecilia Elorm Lekpor

**Affiliations:** ^1^ Department of Medical Laboratory Science Accra Technical University Accra Ghana; ^2^ Department of Medical Laboratory Science KAAF University College Accra Ghana; ^3^ Department of Microbiology Biochemistry and Immunology Morehouse School of Medicine Atlanta Georgia USA

**Keywords:** alcohol, cannabis, hemoglobin, lipid profile, liver, psychiatric

## Abstract

**Background:**

Substance use, particularly cannabis and alcohol consumption, presents a growing public health challenge in Ghana, with significant implications for both mental and physical health. While the adverse psychological effects of these substances have been extensively studied, their biochemical impact remains inadequately explored, particularly in psychiatric settings. The rising prevalence of substance‐use disorders among individuals receiving psychiatric care necessitates a comprehensive evaluation of the physiological consequences associated with cannabis and alcohol use. This study investigated these biochemical effects in patients at Accra Psychiatric Hospital.

**Aim:**

The study sought to determine the impact of the use of cannabis and alcohol on hemoglobin level, lipid profile, and liver function analysis.

**Method:**

This cross‐sectional study at Accra Psychiatric Hospital assessed the impact of cannabis and alcohol use on hemoglobin levels, lipid profile, and liver enzymes among 184 participants. Using stratified purposive sampling, data collection involved structured interviews, and blood analysis. Hemoglobin concentration, lipid profile, and liver enzyme activity were measured using standard laboratory techniques. Statistical analysis was conducted with SPSS and GraphPad Prism, with significance set at *p* < 0.05.

**Results:**

The study compared 92 non‐users with 92 substance users (alcohol‐only, *n* = 21; cannabis‐only, *n* = 50; dual users, *n* = 21). Hemoglobin levels were similar between non‐users and single‐substance users, but significantly higher in dual users compared with non‐users (*p* = 0.017) and alcohol‐only users (*p* = 0.027). Cannabis‐only users had significantly lower total cholesterol than non‐users (4.53 ± 0.98 vs. 5.30 ± 1.23 mmol/L, *p* < 0.001), while LDL‐C was higher in non‐users than in all substance user groups (*p* < 0.05). No group differences were found for HDL‐C, triglycerides, and VLDL. Liver function analysis revealed significantly higher AST in all substance user groups, with dual users recording the highest levels (81.14 ± 72.26 U/L, *p* < 0.001 vs. non‐users). GGT was markedly elevated in alcohol‐only and dual users compared with non‐users (*p* < 0.001). Both direct and indirect bilirubin were significantly higher in all substance user groups (*p* < 0.05), and albumin levels were significantly lower in non‐users than in all substance user groups (*p* < 0.001).

**Conclusion:**

The findings indicate that while single‐substance use of alcohol or cannabis had limited impact on hemoglobin and lipid profiles, dual use was associated with elevated hemoglobin and marked liver enzyme abnormalities. Elevated AST, GGT, and bilirubin in substance users, particularly dual users, suggest potential hepatic stress, warranting targeted public health interventions and monitoring. It is recommended that routine liver function screening be incorporated into healthcare services for individuals with a history of alcohol and cannabis use to enable early detection and management of hepatic impairment.

## 1 Introduction

Cannabis and alcohol are among the most used psychoactive substances worldwide, with significant implications for public health and clinical outcomes [1]. The consumption of these substances is associated with a range of physiological and biochemical changes that can impact vital organ functions, particularly hematological parameters, lipid metabolism, and liver enzyme activity [2]. In Ghana, substance use, including cannabis and alcohol, has become a growing concern, particularly among individuals receiving psychiatric care [3].

Cannabis and alcohol use have been implicated in dyslipidaemia, an imbalance in lipid profiles that increases the risk of cardiovascular diseases [4]. Alcohol, particularly chronic and heavy consumption, has been shown to affect triglyceride and cholesterol levels, potentially leading to atherosclerosis and other cardiovascular complications. Again, alcohol is well‐documented as a hepatotoxic agent, capable of inducing fatty liver disease, hepatitis, and cirrhosis with prolonged exposure [5, 6]. Co‐use of cannabis and alcohol presents a unique challenge in understanding their synergistic or antagonistic effects on liver function [7, 8], which is of particular importance in psychiatric settings where polypharmacy is common.

Given the increasing prevalence of substance use disorders and their potential impact on physiological health, there is a pressing need to generate locally relevant data that can inform clinical practice and policy formulation. This study sought to address this gap by assessing the effects of cannabis and alcohol use on hemoglobin levels, lipid profiles, and liver enzyme activity among patients at Accra Psychiatric Hospital.

## 2 Methodology

### 2.1 Study Design/Site

This study employed a cross‐sectional design to assess the impact of cannabis and alcohol use on hemoglobin levels, lipid profile, and liver enzyme activity among individuals at the Accra Psychiatric Hospital. The Accra Psychiatric Hospital is a leading mental health facility in Ghana. Located in the capital city, the hospital provides specialized care for individuals with psychiatric conditions, including those with substance use disorders. The hospital has a well‐established diagnostic and treatment framework for substance use disorders, allowing for systematic data collection. Its clinical laboratories are equipped to perform routine hematological and biochemical analyses, ensuring reliable assessment of hemoglobin levels, lipid profiles, and liver enzyme activity. Additionally, the structured patient care system at the hospital facilitates the recruitment of participants with documented histories of cannabis and alcohol use, ensuring the study captures relevant clinical and biochemical variations.

#### 2.1.1 Sample Size/Study Population

Ninety‐two drug abusers and 92 non‐drug abusers were recruited for this study. The study population comprised patients diagnosed with substance use disorders involving cannabis and/or alcohol at Accra Psychiatric Hospital.

#### 2.1.2 Sampling Technique

A stratified sampling technique was used to recruit participants for the study.

#### 2.1.3 Inclusion Criteria

Participants must be diagnosed with substance use disorder related to cannabis, alcohol, or both, as confirmed by medical records or self‐reported history. They must be receiving care at Accra Psychiatric Hospital at the time of the study. Participants were included as controls if they had no history of substance use and were healthy.

#### 2.1.4 Exclusion Criteria

Individuals with a history of metabolic disorders such as diabetes, chronic kidney disease, or genetic haemoglobinopathies, which may independently alter hemoglobin levels, lipid profiles, or liver enzymes, are excluded. Patients on medications known to significantly impact lipid metabolism, liver function, or hemoglobin synthesis were also excluded. Patients below 18 years were excluded from the study.

### 2.2 Ethical Considerations

Ethical approval was sought from the Ethical Review Committee of the Medical Laboratory Technology Department, Accra Technical University (Protocol No.: ATU/MLT/ET/01230845B/2024‐2025). Informed consent was also obtained from participants. Study participants were assured of the strict confidentiality and safety of any information they provided for the study. Special measures were taken to ensure participants' autonomy and protect those with impaired decision‐making capacity. Risks were minimized through careful protocol design and continuous monitoring. Cultural sensitivities relating to mental health and substance use were respected, and appropriate support was offered to participants as needed.

### 2.3 Sample Collection

Data collection involved administering structured questionnaires and obtaining blood samples from participants. The questionnaire was designed to gather information on substance use history, frequency, duration, and associated health symptoms, ensuring clarity and ease of comprehension. The questionnaire was administered in the form of a face‐to‐face interviews with participants. Following the questionnaire administration, approximately 4 mL of venous blood was obtained from each participant following standard protocols for phlebotomy. For hemoglobin level assessment, blood samples were collected into an EDTA tube to prevent coagulation, and for biochemical assays, the specimens were collected into a serum separator tube (SST).

### 2.4 Laboratory Analysis

#### 2.4.1 Hemoglobin Level Assessment

Hemoglobin level was measured using the Mindray 5‐Part BC‐5380 Fully Automated Haematology Analyser (Mindray Bio‐Medical Electronics Co. Ltd) for accurate assessment of blood hemoglobin concentration. About 2 mL of venous blood was collected from each participant into an EDTA tube following standard phlebotomy procedures. The amount of hemoglobin in whole blood was expressed in grams per deciliter (g/dL). The normal Hb level for males is 14–18 g/dL; that for females is 12–16 g/dL. When the hemoglobin level is below the lower limit, the patient is said to be anemic [9].

#### 2.4.2 Biochemical Assay for Serum Lipid Profile and Liver Enzyme Assessment

About 3 mL of venous blood was collected from each participant into SST. The serum from each sample was used for the biochemical analysis. Lipid profile assessment included total cholesterol (TC), triglycerides (TGs), high‐density lipoprotein cholesterol (HDL‐C), very low‐density lipoprotein cholesterol (VLDL‐C), and low‐density lipoprotein cholesterol (LDL‐C). Liver enzyme activity was evaluated by measuring alanine aminotransferase (ALT), aspartate aminotransferase (AST), alkaline phosphatase (ALP), and gamma‐glutamyl transferase (GGT) using the DiaSys Respons 910 automated chemistry analyzer manufactured by DiaSys Diagnostic Systems in Germany for concentration readings.

### 2.5 Data Analysis

Data was entered into Microsoft Office Excel 2019 and imported into Statistical Package for the Social Sciences (SPSS) version 27 and GraphPad Prism 8.0 for analysis (GraphPad Prism). Percentages were calculated for categorical variables. Statistical comparisons between subgroups of continuous variables were evaluated by *t*‐test, analysis of variance, and chi‐square test where appropriate. Statistical significance was set at *p* < 0.05.

## 3 Results

### 3.1 Demographic Profile of Study Participants

A total of 184 participants were enrolled in the study, comprising 92 non‐users and 92 substance users. As shown in Table 1, 22.83% (*n* = 21) reported alcohol‐use only, 54.34% (*n* = 50) reported exclusive cannabis use, and another 22.83% (*n* = 21) used both alcohol and cannabis (dual users). Males constituted the majority in all substance use groups, accounting for 90.48% (*n* = 19) in the alcohol‐only group, 88.00% (*n* = 44) in the cannabis‐only group, and 95.24% (*n* = 20) among those using both substances. The majority of cannabis‐only users (*n* = 37, 74.00%) had been using for less than 1 year, while 24.00% (*n* = 12) reported 1–3 years of use, and only 2.00% (*n* = 1) had used for 4–6 years. In the alcohol‐only group, 38.10% (*n* = 8) had used substances for less than 1 year, 33.33% (*n* = 7) for 1–3 years, 23.81% (*n* = 5) for 4–6 years, and 4.76% (*n* = 1) for more than 6 years. Among dual users, 38.10% (*n* = 8) had used substances for less than 1 year, 47.62% (*n* = 10) for 1–3 years, and 14.29% (*n* = 3) for 4–6 years.

**Table 1:** Sociodemographic characteristics, substance use patterns, and perceptions of health impact among participants at Accra Psychiatric Hospital.

	Non‐user (*N* = 92)	Substance user (*N* = 92)		
		Alcohol only (*n* = 21)	Cannabis only (*n* = 50)	Dual usage (*n* = 21)
Gender				
Male	39 (42.39)	19 (90.48)	44 (88.00)	20 (95.24)
Female	53 (57.61)	2 (9.52)	6 (12.00)	1 (4.76)
Age (years)				
18–35	32 (34.78)	7 (33.33)	45 (90.00)	8 (38.10)
36–59	48 (52.17)	14 (66.67)	5 (10.00)	12 (57.14)
> 60	12 (13.04)	0 (0.00)	0 (0.00)	1 (4.76)
Years engaged in substance usage				
<1 year	—	8 (38.10)	37 (74.00)	8 (38.10)
1–3 years	—	7 (33.33)	12 (24.00)	10 (47.62)
4–6 years	—	5 (23.81)	1 (2.00)	3 (14.29)
> 6 years	—	1 (4.76)	0 (0.00)	0 (0.00)
Frequency of substance usage				
Daily	—	3 (14.29)	0 (0.00)	2 (9.52)
Weekly	—	8 (38.10)	4 (8.00)	6 (28.57)
Monthly	—	2 (9.52)	3 (6.00)	5 (23.81)
Occasionally	—	8 (38.10)	43 (86.00)	8 (38.10)
Experience liver dysfunction symptoms				
Yes	—	0 (0.00)	0 (0.00)	0 (0.00)
No	—	21 (100.00)	50 (100.00)	21 (100.00)
Do you believe the substance use has detrimental effect on your health?				
Yes	—	2 (9.52)	0 (0.00)	1 (4.76)
No	—	19 (90.48)	50 (100.00)	20 (95.24)

*Note:* Values, frequencies, and column percentages for non‐users (*n* = 92) and substance users (*n* = 92), categorized into alcohol‐only, cannabis‐only, and dual‐use (both alcohol and cannabis) groups. Percentages are calculated within each column. Liver dysfunction symptoms: Fatigue, dark urine and pale stools, edema, pruritus, jaundice.

### 3.2 Effect of Cannabis and Alcohol Use on Hemoglobin Levels

Table 2 presents the mean hemoglobin level among the non‐substance users and the substance users (alcohol, cannabis, and dual users). Despite non‐significant differences, female drug users were anemic. Among male participants, the mean hemoglobin concentration was 13.76 ± 1.33 g/dL in the non‐user cohort compared to 13.54 ± 1.44 g/dL in the substance user group.

**Table 2:** Comparison of mean hemoglobin levels (g/dL) between non‐users and substance users by gender at Accra Psychiatric Hospital.

Gender	Reference range (g/dL)	Hemoglobin level (g/dL)		*p* Value
		Non‐user	Substance user	
Male	14.00–18.00	13.76 ± 1.33	13.54 ± 1.44	0.409
Female	12.00–16.00	12.39 ± 1.12	11.91 ± 2.16	0.531

*Note:* Values reported are means and standard deviations compared using *t* test to assess statistical difference in hemoglobin levels.

**Figure 1:**
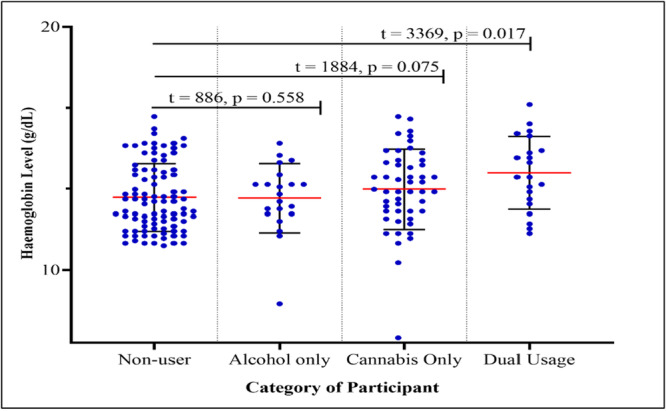
Dot plot showing hemoglobin levels among non‐users, alcohol‐only users, cannabis‐only users, and dual users at Accra Psychiatric Hospital.

Figure 1 illustrates the distribution of hemoglobin concentration among the study participants stratified by category of participant. The mean hemoglobin level recorded among non‐user participants was 12.97 ± 1.39 g/dL, which was not significantly (*U* = 886, *p* = 0.558) higher than the mean hemoglobin level recorded among alcohol users only, 12.94 ± 1.42 g/dL. The mean hemoglobin level recorded among cannabis only users was 13.31 ± 1.64 g/dL, and among participants who are dual users of alcohol and cannabis was 13.98 ± 1.50. Statistically, the difference in mean hemoglobin level between non‐users and cannabis users was not significant (*U* = 1884, *p* = 0.075). However, the difference between non‐users and dual users of alcohol and cannabis was statistically significant (*U* = 3369, *p* = 0.017). Comparatively, the mean hemoglobin level the mean difference between dual users differed significantly from alcohol users (*p* = 0.027) but did not differ significantly from cannabis users' hemoglobin level (*p* = 0.102). More so, the mean difference in hemoglobin level observed between alcohol only users and cannabis only users was also not statistically significant (*p* = 0.347).

### 3.3 Effect of Cannabis and Alcohol Use on Lipid Profile

Table 3 presents the comparison of mean lipid profile parameters between non‐users and the three substance user groups (alcohol‐only, cannabis‐only, and dual users). The mean TC value recorded among non‐users was 5.30 ± 1.23 mmol/L. Alcohol‐only users recorded a slightly lower mean TC (5.16 ± 1.22 mmol/L), while cannabis‐only users had the lowest mean TC (4.53 ± 0.98 mmol/L). The difference between non‐users and cannabis‐only users was statistically significant (*p* < 0.001). The mean HDL‐C value was slightly higher among substance users compared to non‐users. Alcohol‐only users recorded the highest mean HDL‐C (1.76 ± 0.67 mmol/L), followed by dual users (1.69 ± 0.52 mmol/L) and cannabis‐only users (1.62 ± 0.42 mmol/L), while non‐users had the lowest mean HDL‐C (1.56 ± 0.40 mmol/L). LDL‐C was significantly greater than that of alcohol‐only users (2.89 ± 1.18 mmol/L, *p* = 0.012), cannabis‐only users (2.43 ± 0.90 mmol/L, *p* < 0.001), and dual users (2.82 ± 1.23 mmol/L, *p* = 0.009).

**Table 3:** Comparison of mean lipid profile parameters between non‐users and substance user groups at Accra Psychiatric Hospital.

Parameter (mmol/L) [ref range]	Non‐user	Substance user		
		Alcohol‐only	Cannabis‐only	Dual users
TC [0.01–5.20 mmol/L]	5.30 ± 1.23^a^	5.16 ± 1.22	4.53 ± 0.98^a^	5.04 ± 1.40
HDL‐C [0.90–2.30 mmol/L]	1.56 ± 0.40	1.76 ± 0.67	1.62 ± 0.42	1.69 ± 0.52
LDL‐C [0.00–3.40 mmol/L]	3.84 ± 1.11^b^	2.89 ± 1.18^b^	2.43 ± 0.90	2.82 ± 1.23
TGs [0.01–2.30 mmol/L]	1.09 ± 0.60	1.09 ± 0.55	1.14 ± 0.98	0.96 ± 0.46
VLDL [0.01–1.00 mmol/L]	0.38 ± 0.28	0.47 ± 0.24	0.45 ± 0.24	0.43 ± 0.22

*Note:* Values are presented as mean ± standard deviation. Row values with the same superscript (a and b) are significant at *p* < 0.05.

Abbreviations: HDL‐C, high‐density lipoprotein cholesterol; LDL‐C, low‐density lipoprotein cholesterol; TC, total cholesterol; TGs, triglycerides; VLDL, very low‐density lipoprotein.

### 3.4 Impact of Cannabis and Alcohol Use on Liver Enzyme Activity

Table 4 compares liver function parameters between non‐users and substance user groups (alcohol‐only, cannabis‐only, and dual users). The mean total bilirubin recorded among non‐users, alcohol‐only users, and cannabis‐only users was 15.24 ± 37.04, 18.71 ± 17.50, and 16.37 ± 10.93 µmol/L. Direct bilirubin concentrations were significantly higher in alcohol‐only users (7.03 ± 8.59 µmol/L) compared with non‐users (4.47 ± 1.43 µmol/L; *p* = 0.003) and cannabis‐only users (4.61 ± 2.48 µmol/L, *p* = 0.008). Indirect bilirubin was significantly higher in all substance user groups compared to non‐users (7.15 ± 5.69 µmol/L), with alcohol‐only users (11.68 ± 10.74 µmol/L, *p* = 0.032), cannabis‐only users (11.80 ± 8.88 µmol/L, *p* = 0.003), and dual users (14.67 ± 14.82 µmol/L, *p* < 0.001). AST activity in non‐users (21.19 ± 8.83 U/L) was within the reference range (10–40 U/L) but was significantly elevated in all substance user groups. GGT showed the most pronounced elevations, particularly in dual users (95.17 ± 175.47 U/L) and alcohol‐only users (72.47 ± 68.77 U/L) compared to non‐users (29.76 ± 10.99 U/L), with all differences reaching statistical significance (*p* < 0.05).

**Table 4:** Comparison of liver function parameters between non‐users and substance user groups (alcohol‐only, cannabis‐only, and dual users) at Accra Psychiatric Hospital.

Liver enzymes (units)	Reference range	Non‐user (*N* = 92)	Substance users (*N* = 92)		
			Alcohol‐only (*n* = 21)	Cannabis‐only (*n* = 50)	Dual user (*n* = 21)
Total bilirubin (µmol/L)	5–21	15.24 ± 37.04	18.71 ± 17.50	16.37 ± 10.93	20.99 ± 17.07
Direct bilirubin (µmol/L)	0.1–6.8	4.47 ± 1.43^a,b^	7.03 ± 8.59^a,c^	4.61 ± 2.48^c^	6.33 ± 3.28^b^
Indirect bilirubin (µmol/L)	0.1–17.1	7.15 ± 5.69^d,e,f,^	11.68 ± 10.74^d^	11.80 ± 8.88^e^	14.67 ± 14.82^f^
Total protein (g/L)	60–83	53.90 ± 90.59^g^	78.55 ± 5.04	77.80 ± 6.57^g^	76.79 ± 7.68
Albumin (g/L)	35–50	21.14 ± 20.11^h,i,j^	45.81 ± 5.58^h^	46.10 ± 5.32^i^	44.88 ± 5.25^j^
AST (U/L)	10–40	21.19 ± 8.83^k,l,m^	65.83 ± 54.57^k^	54.60 ± 53.79^l,n^	81.14 ± 72.26^m,n^
ALT (U/L)	7–56	34.95 ± 65.63	44.81 ± 41.94	41.98 ± 44.55	46.62 ± 24.79
ALP (U/L)	44–147	81.07 ± 26.24°	84.52 ± 32.52	96.40 ± 36.09°	89.00 ± 27.91
GGT (U/L)	9–48	29.76 ± 10.99^p,q,r^	72.47 ± 68.77^p^	59.62 ± 99.58^q^	95.17 ± 175.47^r^
Globulin (g/L)	20–35	39.93 ± 77.24	31.23 ± 9.30	31.52 ± 4.44	32.19 ± 5.89

*Note:* Values are presented as mean ± standard deviation. Row values with the same superscript (a–r) are significant at *p* < 0.05.

Abbreviations: ALP, alkaline phosphatase; ALT, alanine aminotransferase; AST, aspartate aminotransferase; GGT, gamma‐glutamyl transferase.

## 4 Discussion

Substance abuse, particularly the use of cannabis and alcohol, poses significant health risks, yet its biochemical and hematological effects remain largely unexamined in Ghana. Alterations in hemoglobin levels, lipid metabolism, and liver enzyme activity can indicate serious health complications, making it crucial to understand the physiological consequences of substance use. This study sought to address this gap by examining the impact of cannabis and alcohol consumption on these biochemical markers among patients at Accra Psychiatric Hospital. The findings of this study demonstrate that alcohol and cannabis use, whether in isolation or combination, can influence several biochemical parameters relevant to hematological status, lipid metabolism, and liver function.

The predominance of males among all substance usage categories, particularly in the cannabis‐only and dual‐use groups, reflects patterns reported in both local and international studies. Previous epidemiological research in sub‐Saharan Africa, including Ghana, has consistently documented higher rates of alcohol and cannabis consumption among men compared to women, which is often attributed to socio‐cultural norms, gendered differences in risk‐taking behavior, and greater social tolerance of substance use among males [10, 11]. Age distribution indicated that cannabis use was particularly prevalent among younger adults (18–35 years), whereas alcohol‐only use was more common among older adults (36–59 years). This aligns with studies suggesting that cannabis initiation typically occurs during adolescence or early adulthood, possibly due to peer influence, perceived social acceptance, and the experimental phase of youth [12]. Interestingly, the majority of substance users did not perceive their consumption as detrimental to health. This is consistent with findings from behavioral health studies indicating that individuals often underestimate or discount the health risks of substances they consume, particularly when immediate adverse effects are not evident [13].

With regards to the duration and frequency, majority of cannabis‐only users reported use for less than 1 year, which may indicate recent initiation, potentially associated with increased availability or changing societal perceptions of cannabis and consistent with the findings of Chiu et al. [14]. In contrast, alcohol users, particularly in the older age bracket, demonstrated longer use durations, consistent with studies showing that alcohol consumption often develops into a chronic pattern over years [15, 16]. The predominance of occasional use among cannabis users compared to more evenly distributed usage patterns among alcohol users could reflect differences in the social context of consumption, psychoactive effects, and physiological tolerance development.

Our findings revealed significant increase in hemoglobin levels among dual users compared to non‐users and alcohol‐only users, which may be reflection of the combined effect of both substances, possibly mediated by cannabis‐induced erythropoietic stimulation alongside the absence of severe alcohol‐induced bone marrow suppression in this population, which is consistent with findings of Guzel et al. [17].

This study observed distinct alterations in lipid profile parameters among substance users compared to non‐users, with the patterns varying according to the type of substance used. TC levels were generally higher in alcohol‐consuming groups, particularly in dual users, compared to non‐users. LDL‐C followed a similar trend, with higher levels in alcohol users and particularly in dual users. Alcohol may impair LDL receptor function and increase apolipoprotein B‐containing lipoproteins, thereby elevating LDL‐C levels. This mechanism aligns with the observations of Yu et al. [18], who described increased LDL‐C in chronic alcohol consumers, and whose findings are consistent with this study. HDL‐C levels showed a different pattern. Alcohol‐only users demonstrated elevated HDL‐C compared to non‐users.

TG concentrations were higher in alcohol users, with the most marked elevations seen in dual users. Alcohol is known to promote hepatic TG synthesis while inhibiting their oxidation, leading to hypertriglyceridemia [19]. This occurs partly due to increased acetyl‐CoA availability from ethanol metabolism and partly due to reduced activity of lipoprotein lipase (LPL), the enzyme responsible for hydrolyzing TG‐rich lipoproteins. Chronic alcohol intake can therefore lead to an accumulation of VLDL in circulation, as has been reported elsewhere [20]. The elevated TGs in dual users suggest that cannabis may not counteract, and could potentially exacerbate, alcohol‐induced hypertriglyceridemia. This is supported by findings from Leishman et al. [21], which showed that combined substance use could lead to greater lipid perturbations than either substance alone. Cannabis‐only users in this study did not display significant TG elevations, which may indicate a relatively neutral effect on hepatic TG metabolism in the absence of alcohol.

AST levels were consistently higher in all substance user categories, with the most pronounced elevations observed in dual users. Similar to this study, Acierno et al. [22] and Teschke [23] have reported marked increase in AST associated with alcohol use. The increase observed among alcohol‐containing groups is biologically plausible, as chronic alcohol metabolism via alcohol dehydrogenase (ADH) and the microsomal ethanol oxidizing system (MEOS) generates acetaldehyde and reactive oxygen species (ROS), both of which promote oxidative stress, lipid peroxidation, and mitochondrial injury in hepatocytes [22]. This oxidative damage can increase mitochondrial membrane permeability, leading to the release of AST into the bloodstream. ALT levels did not show significant variation between groups and remained within reference ranges in most participants. ALT is generally considered more specific to hepatocellular injury than AST due to its higher cytosolic concentration in hepatocytes [24]. The relative stability of ALT despite increased AST in alcohol‐only users is consistent with the biochemical signature of early alcoholic liver injury, where mitochondrial damage predominates, producing an AST:ALT ratio greater than 2, as described in several studies [22, 25, 26]. GGT was significantly elevated among all substance users, with the highest mean levels in dual users of alcohol and cannabis. Elevated GGT in the absence of proportional increases in ALT and AST may occur in early or subclinical stages of alcoholic liver disease, which has been well documented in previous reports [26, 27] and is consistent with the findings of this study. Changes in bilirubin fractions were also observed, with both direct (conjugated) and indirect (unconjugated) bilirubin elevated in substance users compared to non‐users. Increases in indirect bilirubin may indicate either enhanced heme breakdown or impaired hepatic uptake and conjugation, whereas elevations in direct bilirubin suggest reduced excretion into bile, possibly due to early cholestatic changes or canalicular transport dysfunction. The findings of this study gain empirical support from the study of Nair et al. [28] and Yang [29] and also support the theory that alcohol can impair bilirubin conjugation by damaging the smooth endoplasmic reticulum. While cannabis smoke exposure has been associated with inflammatory changes in the biliary system in some experimental models. The particularly high values in dual users may indicate greater heme catabolism or impaired hepatic clearance, which could reflect combined hepatocellular and cholestatic effects of concurrent alcohol and cannabis use, which is in line with the findings of López‐Malo et al. [30].

This study has several limitations. First, the cross‐sectional design precludes causal inferences between substance use and observed biochemical changes. The sample was drawn exclusively from a single psychiatric hospital, which may limit generalizability to the wider Ghanaian population. Substance use history was based on self‐report, raising the possibility of recall bias or underreporting due to stigma. Although individuals with major metabolic disorders were excluded, other potential confounders such as diet, smoking, concurrent medications, and psychiatric comorbidities were not fully controlled. Finally, biochemical parameters were measured at a single time point, limiting the ability to capture longitudinal changes. These limitations suggest the need for larger, longitudinal studies incorporating detailed substance use assessments and additional biomarkers to better clarify the mechanisms underlying these associations.

## 5 Conclusion

This study assessed the relationship between alcohol and cannabis use and selected hematological and biochemical parameters among patients at the Accra Psychiatric Hospital. The results showed that hemoglobin concentrations were largely comparable between non‐users and most substance user groups, with the exception of dual alcohol and cannabis users, who exhibited significantly higher values. Evaluation of lipid profiles revealed that cannabis‐only users had significantly lower TC and LDL cholesterol compared with non‐users, while HDL cholesterol and TGs remained within the normal range across all groups. Liver function analysis identified marked elevations in AST and GGT in alcohol users and dual users, consistent with established patterns of alcohol‐related hepatocellular injury and enzyme induction. These findings demonstrate that cannabis and alcohol use are associated with measurable alterations in lipid metabolism and liver enzyme activity, with some effects more pronounced in individuals who use both substances concurrently.

## 6 Recommendations

There is a need for targeted health education campaigns to raise awareness about the biochemical and hematological consequences of alcohol and cannabis use, particularly when these substances are consumed together. Public messaging should emphasize the potential long‐term risks to liver health, cardiovascular function, and blood parameters. Community‐based interventions promoting the reduction or cessation of alcohol and cannabis use could help lower the associated biochemical and hematological risks. These interventions should be supported by counseling services and access to rehabilitation programmes where needed. Healthcare providers should consider regular screening of liver function tests, lipid profiles, and hematological indices in individuals with a history of alcohol or cannabis use. Early detection of abnormalities may allow for timely interventions to prevent progression to more serious health conditions.

## Author Contributions

**Felix Abekah Botchway:** conceptualization, investigation, writing – original draft, methodology, validation, visualization, writing – review and editing, formal analysis, project administration, data curation, supervision, resources.

## Funding

The authors received no specific funding for this work.

## Conflicts of Interest

The authors declare no conflicts of interest.

## Transparency Statement

The lead author Felix Abekah Botchway affirms that this manuscript is an honest, accurate, and transparent account of the study being reported; that no important aspects of the study have been omitted; and that any discrepancies from the study as planned (and, if relevant, registered) have been explained.

## Data Availability

The data that support the findings of this study are available from the corresponding author upon reasonable request. The data used in this study are not publicly available due to confidentiality of information; however, the data are available from the corresponding author on a reasonable request basis.
